# Access to care among Medicaid and uninsured patients in community health centers after the Affordable Care Act

**DOI:** 10.1186/s12913-019-4124-z

**Published:** 2019-05-08

**Authors:** Veri Seo, Travis P. Baggett, Anne N. Thorndike, Peter Hull, John Hsu, Joseph P. Newhouse, Vicki Fung

**Affiliations:** 1 Health Policy Research Center, Mongan Institute, Massachusetts General Hospital, 100 Cambridge Street, Ste. 1600, Boston, MA 02114 USA; 2 Division of General Internal Medicine, Massachusetts General Hospital, 100 Cambridge St., Ste. 1600, Boston, MA 02114 USA; 3 Department of Medicine, Harvard Medical School, 25 Shattuck St, Boston, MA 02115 USA; 4 Institute for Research, Quality, and Policy in Homeless Health Care, Boston Health Care for the Homeless Program, 780 Albany St, Boston, MA 02118 USA; 5 The Becker Friedman Institute, University of Chicago, 1126 E 59th St, Chicago, IL 60637 USA; 6 Department of Health Care Policy, Harvard Medical School, 180 Longwood Ave, Boston, MA 02115 USA; 7 Department of Health Policy and Management, Harvard T.H. Chan School of Public Health, 677 Huntington Ave, Boston, MA 02115 USA; 8 Harvard Kennedy School, 79 John F. Kennedy Street, Cambridge, MA 02138 USA

**Keywords:** Medicaid, Uninsured, Community health centers, Safety-net, Access to care

## Abstract

**Background:**

The Affordable Care Act expanded Medicaid and increased federal funding for Community Health Centers (CHCs). To examine the role of Medicaid coverage on care patterns for those with available safety net care, we assessed differences in access to care for CHC patients with continuous Medicaid coverage vs. gaps in insurance coverage in the last year.

**Methods:**

We used data on adult respondents from the 2014 Health Center Patient Survey (*N* = 1720) with continuous Medicaid coverage vs. those with some period without insurance coverage in the last 12 months. We examined reported need for any medical care, mental health care, prescription drugs, dental care, and referrals for care outside of the CHC in the last 12 months, and reports of being delayed or unable to get needed care by insurance status. We used logistic regression to assess the association between insurance status and care access, adjusting for patient characteristics.

**Results:**

Patients with insurance gaps and continuous Medicaid coverage reported similar levels of need for most types of care in the last 12 months, but those with insurance gaps were significantly more likely to report having difficulty obtaining medical care, prescription drugs, dental care, and completing outside referrals. Of those with incomplete referrals for care outside of the CHC, patients with insurance gaps were more likely than those with continuous Medicaid to cite cost or insurance-related reasons for not following up (70% vs. 19%, *p* < 0.01).

**Conclusions:**

Having continuous Medicaid coverage appeared to mitigate barriers to care for CHC patients compared to having intermittent or no insurance coverage over the last year. Policies that increase disruptions in Medicaid coverage could adversely impact access to care, even among those with available safety net care.

## Background

Increases in the availability of safety net care and insurance coverage could improve access to medical care for low-income populations. The Affordable Care Act (ACA) increased federal funding for Community Health Centers (CHC), which provide care to underserved areas and populations, and also expanded Medicaid eligibility to adults with incomes less than 138% of the federal poverty level (FPL). To date, fourteen states have not yet adopted Medicaid expansion.

CHCs provided care to 27 million patients in 2017 and continue to be an important source of care post-ACA for low-income patients. Following Medicaid expansion, the share of CHC patients with Medicaid grew from 38 to 49% between 2010 and 2017 [1]. Some evidence suggests that gaining Medicaid coverage increases access to primary care and reduces financial barriers to care [2–4]. Studies have also found that there were greater increases in visits to CHCs and improvements in some measures of quality of care provided by CHCs after 2014 in states that expanded vs. did not expand Medicaid [5–7]. Less is known, however, about how having Medicaid coverage compared with no insurance influences care for patients with regular access to safety net care, such as CHCs, which provide care regardless of patients’ ability to pay. In addition to primary care, many CHCs also provide mental health care, dental care, and substance use disorder treatment.

In this study, we used national survey data to examine access to different types of care for CHC patients in 2014, including medical care, mental health care, prescription drugs, dental services, and referrals for care outside of the health center. We compared access to care for those who were continuously covered by Medicaid vs. those with gaps in insurance coverage in the last year.

## Methods

We used publicly available data from the 2014 Health Center Patient Survey (HCPS), a nationally representative survey of CHC patients sponsored by the Health Resources and Services Administration (*N* = 7002; response rate = 91.4%) [8]. The HCPS was fielded in person at federally funded health centers between October 2014–April 2015 among patients with at least one prior visit to the CHC in the last 12 months. All data were deidentified and the study was exempted by the Institutional Review Board.

We limited our study to adult respondents ≥18 years old seeking care at CHCs (*N* = 3172), excluding respondents seen at health centers serving specialty populations (i.e., Health Care for the Homeless, Migrant Health Centers, and Public Housing Primary Care; 9% of the weighted sample of adult respondents). Because the survey measured care access in the 12 months prior to the time of the survey, we limited our Medicaid sample to those continuously enrolled in Medicaid for ≥12 months (*N* = 750; 69% of respondents who reported having Medicaid at the time of the survey). To identify respondents who were uninsured for any period in the last 12 months, we included those who reported being uninsured at the time of the survey (*N* = 598) as well as those currently insured who reported being uninsured at some point in the last 12 months (*N* = 372). The HCPS did not collect information on the length of time respondents were uninsured.

Respondents reported if they needed any medical care, mental health care, prescription drugs, and dental care in the last 12 months, and, if so, whether they were delayed or unable to get the needed care. Additionally, respondents reported if they were referred for care outside of the CHC in the last 12 months, and, if so, whether they had followed up on their most recent referral. If respondents had not yet followed up, they were asked to select the main reason for the incomplete referral. We classified these reasons into three categories: cost/insurance (e.g., could not afford care, insurance company would not approve/doctor would not accept/no insurance); logistics (e.g., no time off work, problems getting to appointment); and other. Among those with incomplete referrals, 63 respondents reported that their appointment was pending, still being scheduled, or that they were just referred at today’s visit; we excluded these respondents from the analysis of incomplete referrals. Findings were similar in sensitivity analyses that included these respondents.

We compared the demographic and health characteristics of CHC patients with Medicaid to those who were uninsured for any period in the last 12 months, including patient age, gender, race/ethnicity, whether a language other than English was spoken at home, education, income, self-rated health status, disability status, and mental health status. The HCPS also included information on the urban/rural status of the respondents’ CHC and whether the respondent reported the CHC to be their usual source of care. We determined disability status based on reported difficulty in six domains of functionality, using the Centers for Disease Control and Prevention’s standard disability questions [9]. We determined mental health status by respondents’ Kessler Psychological Distress Scale (K6) scores, with a score ≥ 13 indicating a high degree of psychological distress [10].

We compared the percentage of respondents who reported needing the different types of services, and the percentage who reported having any difficulty obtaining that care for those with continuous Medicaid vs. insurance gaps. We used multivariate logistic regression models to examine the association between insurance status and difficulty obtaining care, adjusting for the patient characteristics described above. We applied sampling weights to account for the complex survey design.

## Results

In this sample of CHC patients, the majority were female (68.1%), had a high school education or less (65.2%), and household income less than the federal poverty level (61.2%). Nearly half reported being of non-White race/ethnicity; those who were uninsured for any period in the last 12 months vs. those with continuous Medicaid coverage were more likely to be White (46.4% vs. 52.4%) or Hispanic (28.9% vs. 20.5%), and less likely to be Black (14.1% vs. 25.5%, *p* < 0.05, Table 1). In addition, those who were uninsured vs. Medicaid-covered were more likely to speak a language other than English at home (32.0% vs. 19.8%) and visit CHCs in rural areas (52.4% vs. 36.8%, *p* < 0.05).

**Table 1:** Demographic and health traits by insurance status

	Total (*N* = 1720)	Continuous Medicaid coverage last 12 months (*N* = 750)	Uninsured for any period in last 12 months (*N* = 970)	*p*-value
Demographics				
Age group				0.12
18–44	61.4	66.0	58.5	
45–64	37.7	32.9	40.8	
65+	0.9	1.1	0.7	
Male	31.9	26.5	35.2	0.05
Race/ethnicity				< 0.01
White	50.1	46.4	52.4	
Black	18.5	25.5	14.1	
Asian	1.8	2.6	1.3	
Hispanic	25.7	20.5	28.9	
Other	4.0	5.0	3.3	
Another language at home	27.3	19.8	32.0	< 0.01
Education				0.63
< High School (HS)	34.4	34.8	34.2	
HS or GED	30.8	33.5	29.2	
> HS	34.3	31.3	36.2	
Other	0.4	0.4	0.4	
Household income				0.17
≤ 100% of the Federal Poverty Level (FPL)	61.2	66.9	57.6	
101–138% FPL	16.5	15.9	16.9	
139–199% FPL	11.5	8.1	13.7	
200% + FPL	10.8	9.1	11.8	
Rural	49.3	36.8	52.4	< 0.01
Health indicators				
Health status				0.64
Excellent/Very good	21.9	20.3	22.8	
Good	36.7	39.2	35.1	
Fair/Poor	41.5	40.4	42.1	
Disabled	43.5	41.0	45.0	0.39
Psychological distress (K6 ≥ 13)	38.0	30.2	42.8	< 0.01
CHC is usual source of care	81.7	81.9	81.6	0.94
Current insurance status				
Medicaid		100.0	24.9	
Medicare or dual-eligible		–	1.2	
Private (group and non-group)		–	5.5	
Other/unspecified public or private		–	1.6	
Uninsured		–	66.8	

Notes: Those uninsured for any period in the last 12 months include respondents who reported being uninsured at the time of the survey and currently insured respondents who reported being uninsured at any point in the last 12 months. Those with Medicaid include respondents who reported having Medicaid coverage in each of the last 12 months. The current insurance status distribution excludes respondents who answered “don’t know” or refused to answer (0.1% of the uninsured sample)

Most respondents (81.7%) in both groups reported that the CHC was their usual source of care. About 40% of both groups reported being in fair or poor health, having a functional disability, or had K6 scores indicating psychological distress; psychological distress was more common among those with insurance gaps in the last 12 months vs. continuous Medicaid (42.8% vs. 30.2%, p < 0.05). Among those who were uninsured sometime in the last 12 months, 33% were currently insured at the time of the survey, with Medicaid being the most common source of insurance (25%).

**Fig. 1:**
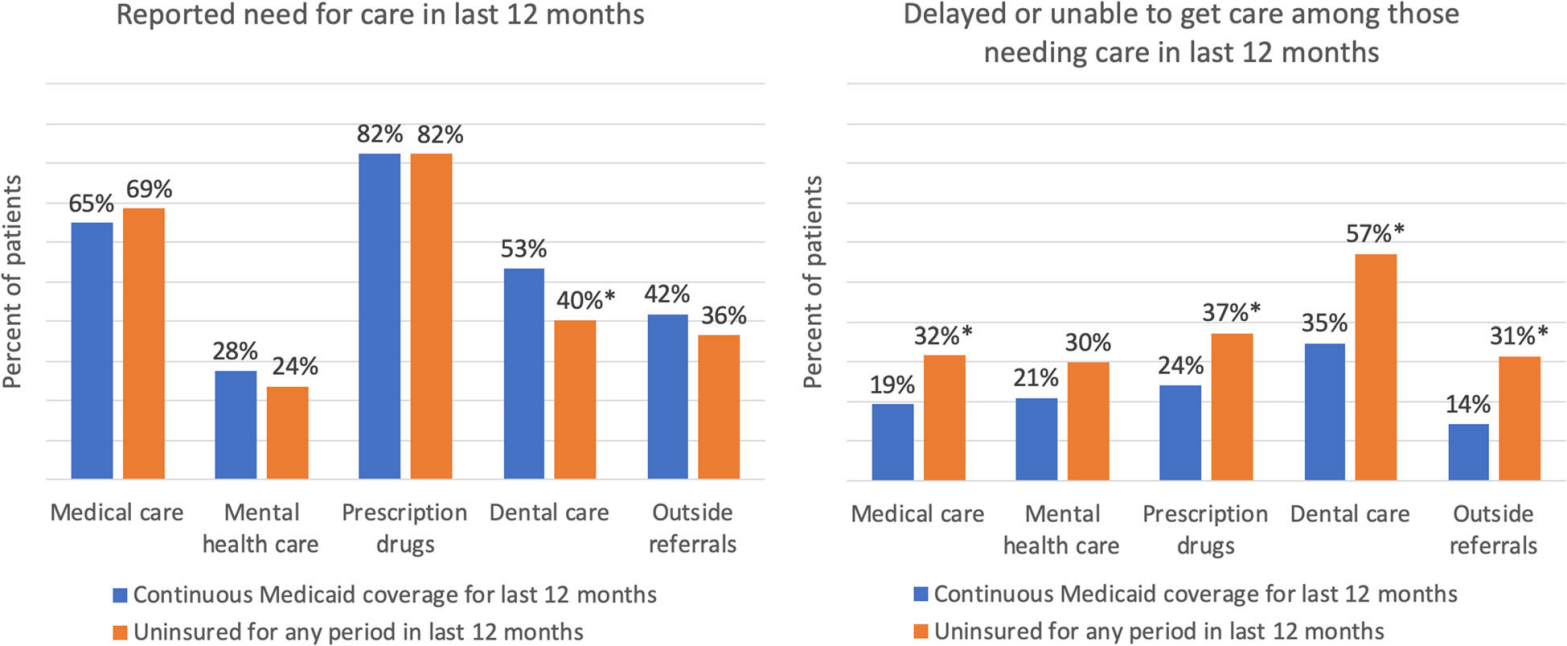
Need for care and reported difficulties in obtaining care by insurance status. Notes: Those uninsured for any period in the last 12 months include respondents who reported being uninsured at the time of the survey and currently insured respondents who reported being uninsured at any point in the last 12 months. Those with Medicaid include respondents who reported having Medicaid coverage in each of the last 12 months. Percentages are weighted to account for the survey design. * Significant difference (*p* < 0.05) between Medicaid vs. uninsured groups

About two-thirds in both groups reported needing medical care in the last 12 months (Fig. 1). Reported needs for mental health care, prescription drugs, and referrals for care outside of the CHC were also similar between the groups.

Among those needing medical care, 27% reported being delayed or unable to obtain care. Those uninsured in the last 12 months were more likely to report difficulties in obtaining medical care, prescription drugs, dental care, and outside referrals vs. those with continuous Medicaid coverage (*p* < 0.05, Fig. 1). Differences in obtaining needed mental health care were not significant. After adjusting for individual demographic and health traits, those with insurance gaps vs. continuous Medicaid were significantly more likely to have difficulty obtaining medical care, prescription drugs, and dental care, and to have not completed referrals for care outside of the CHC (Table 2).

**Table 2:** Adjusted odds of reporting difficulty obtaining needed care for CHC patients who were uninsured for any period in last 12 months vs. patients continuously enrolled in Medicaid in the last 12 months

Type of care	Odds Ratio	95% CI
Delayed/unable to get medical care	2.0	1.1–3.8
Delayed/unable to get mental health care	1.6	0.7–3.6
Delayed/unable to get prescription drugs	1.9	1.1–3.3
Delayed/unable to get dental care	2.4	1.3–4.6
Did not follow up on most recent referral	3.7	1.7–7.9

Notes: Those uninsured for any period in the last 12 months include respondents who reported being uninsured at the time of the survey and currently insured respondents who reported being uninsured at any point in the last 12 months. Those with Medicaid include respondents who reported having Medicaid coverage in each of the last 12 months. Logistic regression models adjust for respondent demographic and health characteristics in Table 1. All analyses are weighted to account for the survey design

Among those with incomplete referrals, 70% of those with insurance gaps, compared to 19% of those with continuous Medicaid, cited cost or insurance-related barriers as the main reason (*p* < 0.01, Table 3). Those with continuous Medicaid were more likely to cite logistical barriers as the reason.

**Table 3:** Reasons for not following up on referral, by insurance status

	Continuous Medicaid coverage last 12 months (*N* = 34)	Uninsured for any period in last 12 months (*N* = 90)	*p*-value
Cost/insurance barriers	18.9%	69.8%	< 0.01
Logistical barriers	60.0%	13.8%	
Other	21.1%	16.4%	

Notes: The number of respondents is unweighted; percentages are weighted to account for survey the design. Responses are reclassified into categories and exclude patients with pending appointments, who were waiting for the referral, needed to reschedule their appointment, or were referred at the time of the survey. Those uninsured for any period in the last 12 months include respondents who reported being uninsured at the time of the survey and currently insured respondents who reported being uninsured at any point in the last 12 months. Those with Medicaid include respondents who reported having Medicaid coverage in each of the last 12 months

## Discussion

Among CHC patients, we found that those with continuous Medicaid coverage and those who were uninsured sometime in the last year had similar levels of need for most types of care. However, patients reporting disruptions in insurance coverage had greater difficulty obtaining care compared to those with continuous Medicaid coverage.

CHC revenues from Medicaid reimbursement have grown post-ACA. In addition, the ACA created the Community Health Center Fund (CHCF), which provided $11 billion in mandatory funding for CHCs between 2011 and 2015 [11]. These funds accelerated recent growth in the number of health center delivery sites, staffing, and service provision; between 2011 and 2016, the number of CHC sites grew by 50% and the number of patients served by 33% [12]. The CHCF, which now comprises 70% of federal funding for CHCs, was recently reauthorized through the Bipartisan Budget Act of 2018, but only through 2019 [13].

All CHCs provide primary care services, with 89% providing mental health services, 81% providing dental services, and 35% providing substance use treatment in 2017 [1]. However, uninsured and Medicaid patients seeking care at CHCs reported high levels of medical need and complexity, which necessitated referrals outside of the CHC. Those with gaps in coverage were significantly less likely to complete these outside referrals compared to those with continuous Medicaid coverage, with most uninsured patients citing the cost of care or lack of insurance as the reason. Although the survey did not collect information on the specific type of care needed, these referrals are likely to be for specialty care not provided in the CHC; forgoing such care may result in worse outcomes.

Notably, among those who were uninsured for some period in the last 12 months, nearly a quarter currently had Medicaid, but still faced greater difficulties accessing care compared to those with continuous Medicaid enrollment. In January 2018, the Centers for Medicare and Medicaid Services reversed guidance for Section 1115 waivers to allow states to impose work requirements as a condition for Medicaid eligibility. Fifteen states have submitted such waivers with seven approved as of April 2019 [14]. Some states are also including other provisions, such as increasing cost-sharing requirements or stipulating lock-out rules, which could impact Medicaid eligibility and increase the number of enrollees with disruptions in coverage. Our findings suggest that policies that increase Medicaid churn or disruptions in coverage could result in greater barriers for obtaining needed medical care, even among those with access to safety net care at CHCs.

This study has limitations. This is a cross-sectional study and there could be unmeasured confounding, although we were able to adjust for a number of sociodemographic and health-related traits. The comparison groups were similar with respect to a number of key traits, including age, education, household income, and health and functional status; however, those with gaps in insurance coverage were more likely to speak a non-English language at home, live in rural areas, and have psychological distress, which could contribute to additional barriers in accessing care.

The survey also did not capture how long respondents had gaps in insurance coverage or the reason for their lapse in coverage. The HCPS sample includes CHC patients with at least one prior visit and may not be generalizable to those who use CHCs more sporadically. We were not able to link patient responses with information on CHC service availability or whether respondents were directly affected by Medicaid expansion. Lastly, reasons for having difficulty obtaining care were only available for incomplete referrals, but not the other types of care in the survey.

## Conclusion

There is continued debate about how best to provide and finance care for low-income Americans. Having continuous Medicaid coverage appeared to mitigate barriers to care among CHC patients compared to having intermittent or no insurance coverage, highlighting the complementary roles of the Medicaid and CHC programs for underserved populations. Policies that increase Medicaid churn could adversely impact access to care, even among those with available safety net care.

## Abbreviations

ACA: Affordable Care Act
CHC: Community Health Centers
CHCF: Community Health Center Fund
HCPS: Health Center Patient Survey
